# Social Media Fact-Checking: The Effects of News Literacy and News Trust on the Intent to Verify Health-Related Information

**DOI:** 10.3390/healthcare11202796

**Published:** 2023-10-22

**Authors:** Ines Kožuh, Peter Čakš

**Affiliations:** Faculty of Electrical Engineering and Computer Science, University of Maribor, 2000 Maribor, Slovenia; peter.caks@um.si

**Keywords:** fact-checking, news literacy, trust, social media, misinformation, fake news, pandemic, health crisis

## Abstract

The recent health crisis and the rapid development of Artificial Intelligence have caused misinformation on social media to flourish by becoming more sophisticated and challenging to detect. This calls upon fact-checking and questions users’ competencies and attitudes when assessing social media news. Our study provides a model of how fact-checking intent is explained by news literacy and news trust to examine how users behave in the misinformation-prone social media environment. Structural equation modeling was used to examine survey data gathered from social media users. The findings revealed that users’ intent to fact-check information in social media news is explained by (1) news literacy, such as the awareness of various techniques used by creators to depict situations about COVID-19; (2) news trust, in terms of the conviction that the news contains all the essential facts; and (3) intent, such as an aim to check information in multiple pieces of news. The presented findings may aid policymakers and practitioners in developing efficient communication strategies for addressing users less prone to fact-checking. Our contribution offers a new understanding of news literacy as a sufficient tool for combating misinformation, which actively equips users with knowledge and an attitude for social media news fact-checking.

## 1. Introduction

In the recent health crises, social media have emerged as the key source for finding and transmitting health-related information. This was the case during the recent COVID-19 pandemic [1,2,3,4,5,6] and the last Ebola outbreak [7]. Anxiety and uncertainty in society aroused by the nature of the diseases and continuous breakthroughs in the field [8] have created a suitable environment for spreading misinformation. Individuals have been surpassing media outlets to become critical players in information diffusion [9]. In addition, Artificial Intelligence (AI) has also recently been playing a noticeable role in the production and dissemination of misinformation [10]. Machine and Deep Learning have become ready-made tools, available to individuals in the comfort of their homes [11] through AI-powered chatbots [12] (p. 2), enhanced features, and deep fake tools, as advertised on social media [13] (p. 2).

Accordingly, social media have become a misinformation-prone environment [14,15,16] with favorable conditions for misinformation to flourish due to the lack of gatekeeping and regulations [17,18]. As the spread of public health misinformation could result in significant harm to (mental) health [3,19,20] or even life [21], combating misinformation should be one of the top concerns in media content today.

Misinformation and disinformation are often named fake news [22,23,24]. Namely, misinformation is false information that does not necessarily intend to mislead [25,26], and it is usually spread by a communicator who does not know that the information is false [24] or is willing to share such information, even if (s)he cannot verify its accuracy [22]. Though not necessarily ill-intentioned, other users may still perceive it as malicious and build false beliefs based on the provided material [26]. On the contrary, disinformation is a piece of information intended to mislead. Knowing it to be false, a communicator promotes it as true [8,26] to create an unstable environment [22].

In the case of COVID-19, it has frequently been difficult to distinguish between misinformation and disinformation, as it has been impossible to identify whether communicators of such information intend to deceive [26,27]. Misinformation about COVID-19 has been challenging to define due to its multi-layered nature, different perceptions among various subgroups of the population [28], and ongoing new scientific findings replacing existing ones [26]. These conditions have also influenced the sharing of misinformation about COVID-19 [23] due to low digital health literacy and scarce general medical knowledge in the background of vaccination and disease spreading [29,30,31]. Misinformation in the context of COVID-19 can, thus, be defined as any messages that are in disagreement with the strongest evidence currently available regarding COVID-19. If they are disputed, they are likely to remain uncorrected [8].

The situation urges fighting against misinformation, which necessitates ferocious efforts and extensive collaboration [31]. There have been several attempts to combat misinformation at the regulatory, community, and corporate levels. At the regulatory level, in 2018, the European Commission released the “Action Plan against Disinformation” [32], followed by the EUvsDisinfo website [33]. At the community level, public discussions have been heavily oriented toward the best practices of misinformation dismantling, with a media panel organized by the European External Action Service (EEAS) in 2023 [34]. At the corporate level, the advertising sector and online platforms came to an agreement on a “Code of Practice on Disinformation” [35] to combat false information. Thus, major tech companies introduced warnings about false information, automated fact-checking, and tools for users to tag false news manually [36,37,38]. And social media industry leaders, some now united under Meta Platforms, Inc., expressed their agreement to fight misinformation with the release of a joint statement on combating fake news in 2020 [39].

The available research does not uniformly postulate how to efficiently tackle misinformation. While some studies indicate that warnings about misinformation, messages about biases, and text annotation improve consumers’ awareness [8,40,41,42,43,44], others believe they are only effective in the short term [45] or not effective at all [46]. Warnings may have a meaningful immediate impact but may not maintain this effect in the long term, as the misinformation and correct information become more and more dissociated over time [45]. This is partly due to human memory functions. Once the arbitrated misinformation resides inside our memory, it simply coexists next to the correction forever, thus forming a mixture of new knowledge, which can even result in the return of the false belief [42]. Taking this phenomenon in terms of prior knowledge, if news includes false beliefs it increases the level of trust, even though the news is untrustworthy [47]. Finally, education at various levels and awareness campaigns cannot provide a complete solution independently [48]. Enforced regulation and penalties imposed on media platforms disseminating misinformation are deemed necessary [42].

Recent research provides possible solutions for the above-mentioned challenges by highlighting various literacy-oriented approaches. It recognizes information literacy [49], digital literacy [50,51,52], social media literacy [53], and news literacy [54] as some of the primary measures for tackling misinformation [36,54,55,56,57,58]. It is relevant to the present study that news literacy equips users with knowledge of news production, consumption, and dissemination and the news environment [54]. It allows users to become fact-checkers, as they may already have the potential to respond actively to misinformation [56,59]. Accordingly, users can self-assess the reliability of information, share high-quality information regarding COVID-19 [36,54,60,61], and make well-informed decisions based on media literacy knowledge supported by (AI) tools for fact-checking [62].

To understand and combat false information holistically, we must first understand what drives users to engage or disengage with misinformation. Digital literacies often overshadow the power of news literacy; however, their newer definitions offer more range for practical implementation [63]. With many studies focusing primarily on technological solutions for fake news in health crises, much is still to be examined at the level of users and their perception of news, which influences their decision making. Thus, our study aims to fill a deficit in misinformation research by providing a new theoretical model that positions news trust [64,65,66] at the core of the relationship between antecedent news literacy [54,56] and users’ fact-checking intent [57], especially during health crises.

The current paper is structured as follows: Section 2 introduces the theoretical framework, the research model, and an overview of the findings related to the main concepts. Section 3 presents the procedure, measuring instrument, sampling, and data analysis, while Section 4 provides the statistical results of the relationships between news literacy, news trust, and fact-checking intent. Section 5 explains empirical insights concerning the existing research. In Section 6, we follow up with conclusive remarks and provide recommendations for further research.

## 2. Conceptual Framework

In the following section, we operationalize vital concepts and explain research questions for the proposed research model on how news literacy and news trust may affect users’ intention to fact-check information in the news about COVID-19. The model focuses on COVID-19 news shared on social media, not by established news organizations but by ordinary users, as they inhabit a space that is not yet regulated, without professional editors or the ethical guidelines enforced in traditional media.

Social media users are content creators and consumers, often relying on the information shared within their network, built up by their acquaintances, whom they trust [65]. With social media becoming the prime source of information during the pandemic [67,68,69], the influence associated with journalistic professionals and, consequently, the trust in the credibility of their work changed significantly. The individual users with large online followings that were the most vocal about the disease and its handling transformed into opinion leaders who could shape the perception of their followers and influence their news trust [70].

Accordingly, we formulated the following research questions:

RQ1: How does news literacy explain fact-checking intent?
RQ2: What role does news trust play in explaining the relationship between news literacy and fact-checking intent?

### 2.1. Key Concepts for Misinformation Dissemination Susceptibility

#### 2.1.1. Fact-Checking Intent

Misinformation malice led to an increase in non-regulatory initiatives such as fact-checking. This refers to recognizing scientific facts and perspectives in combating sensationalist bias and the verification of content [71], which has been described as the starting point of the efforts combating false online health and science information [72,73]. Fact-checking can be understood at the macro and micro levels.

At the macro level, significant tech and social media companies had already taken measures to combat false news before the pandemic [73]. The measures focused on hiring fact-checkers and tagging or removing suspicious or fraudulent posts [74]. These actions are automated and canbe performed manually by users at the micro level. For instance, in the case of COVID-19, Facebook has been removing any claims and conspiracy theories (as labeled by global health authorities) that may cause immediate harm to users [75]. These claims are first recognized by their Artificial Intelligence model and then sent to paid fact-checking contractors as a two-step misinformation detection process. On the contrary, Twitter was initially less communicative about misinformation debunking in their official stance but changed course in 2021 with the Birdwatch community-based model of fact-checking [76].

At the micro level, users may benefit significantly from measures taken to combat false news at the macro level. They may use online fact-checking tools, specialized web platforms that examine rumors and health and political claims that appear primarily on social media [77]. Renowned examples include Oštro.si (Slovenia), Reuters Fact Check, FactCheck.org, etc. [78].

However, more than fact-checking was needed in the comprehensive tackling of misinformation related to COVID-19 [8,79]. Firstly, it was frequently insufficient or impossible to articulate “the facts” about COVID-19 due to ongoing research about the nature of the disease and continuous new findings [8,26]. This led the social media space to be flooded every day with interchanging information about COVID-19, where misinformation had become more sophisticated and challenging to detect. Misinformation has, thus, been slipping through automated [80] and professional fact-checking [22].

This led us to comprehensively examine how users check facts by themselves, following a three-part approach [60,81,82,83]: (1) seeking information from other sources, (2) seeking information in other users’ opinions, and (3) using online tools for fact-checking. Namely, existing research [84] revealed a correlation between users’ positive attitudes towards fact-checking and their intent to examine information critically before sharing it further due to an acknowledgement that they may spread false information to other users, leading to false narratives and beliefs. Thus, in the current study, we focused on fact-checking intent and included it as one of the crucial concepts in our research model.

#### 2.1.2. News Literacy

The term news literacy has a variety of similar definitions and interpretations, and they can all recognize news and analyze and evaluate information critically [85]. News literacy, according to Ashley [86], represents a variety of abilities and the expertise needed to traverse the complex media landscape successfully [86]. Similarly, it can be understood as an understanding of the social and personal processes involved in creating, sharing, and consuming news, as well as the ability to exert some control over these processes [54]. It is multidimensional, so assessing individual news literacy levels is difficult. Theoretical subcomponents, such as (1) authors and audiences, (2) messages and meanings, and (3) representation and reality, are consequently employed to understand the process behind the creation, delivery, positioning, and influence of news messages. Ashley [86] explained these subcomponents as an understanding of how authors target audiences, how production techniques and embedded value convey meaning, and how media representations influence perceptions of reality [86].

In social media, we can understand news literacy as a collection of practical, intellectual, and emotional abilities that allow the creation of content or the detection of fake news posts [87]. Additionally, a news-literate user understands how social media work and employs various tactics to assess social media news, such as validating content, sources, and forms and relying on judgments of known and unknown others [63].

Several authors have recognized the need to investigate (news) literacy concerning the spread of misinformation [36,54,55,56,57]. Researching the impact of existing media literacy skills on tackling misinformation was crucial in the context of health crises [54] and beyond [56,88]. However, there are discrepancies when it comes to utilizing one’s knowledge and skills related to news production in everyday life [63], especially on social media, where low (digital) literacy leads to lower misinformation detection [50,51,53]. Even though social media users may have news literacy knowledge and skills, they do not necessarily apply them in practice [63]. We, thus, believe that it is necessary to consider users’ attitudes towards content in order to predict attitudes or fact-checking intent better. Accordingly, we will be able to better understand users’ ability to apply news literacy tactics in practice, which may, consequently, contribute to more efficient tackling of misinformation. This led us to consider news literacy as another critical concept in our study, which was added to our research model.

#### 2.1.3. News Trust

News trust is an individual’s perception and evaluation of news media [89]. The development of trust in media can occur at the level of content through the credibility of news media, those delivering the content with trust in an institution, and media ownership through the believability of news media organizations [66,90]. In this study, we consider news trust at the level of the attitude toward the content.

Three key dimensions of the attitude toward the content are trust in the selectivity of topics, facts, and the accuracy of depictions [89]. We aimed to examine users’ perceptions, or at least how much they believed a news message was an accurate depiction of reality in a health crisis.

People use social media to retrieve information [91]. Besides following established media outlets, they have a network of friends where they find “opinion leaders” who shape their news trust significantly [65]. This network also affects the perception of the credibility of the information they receive, as does the social media platform through which they receive it [90]. On the one hand, users in an individual’s network can be qualified to provide accurate information; on the other hand, they can produce, share, or validate false information [92]. This applies to COVID-19 as well. Social media exposure was linked with misperceptions about COVID-19, and the reverse was true for news media [93,94].

This led us to recognize news trust as a final key concept in our study, where we considered to what extent users believe that the news on social media contains all the essential details and viewpoints regarding global health-related cases.

### 2.2. Research Questions

#### 2.2.1. RQ1: Relationship between News Literacy and Fact-Checking Intent

When people engage with COVID-19 news on social media, it depends mainly on how they perceive their practical, intellectual, and emotional abilities to tackle the news, in the way of creating the content, assessing the technicalities of the platform that offers the information, and detecting misleading information [15,63,87]. However, it is not yet apparent how much users’ news literacy affects their trust in news and their intent to fact-check information as well as outside implications for politics, voting behavior, and democracy [57], and (news) trust is included as an essential factor affecting misinformation assessment [56].

A previous study [46] revealed that even those with a high level of news literacy might not apply their knowledge to differentiate between high- and low-quality information. The differentiation of information may result from users’ intent to check information in the news they engage with in different ways. Notably, users may intend to seek other sources of information and other users’ opinions and may intend to verify information with specialized online tools for fact-checking. Additionally, when people transition from reading news on social media towards more complex actions, prior knowledge of the news issue takes the leading role instead of personal traits [47]. This phenomenon calls for research on how news literacy translates into behaviors that may affect the tackling of misinformation [54,63], i.e., fact-checking intent.

#### 2.2.2. RQ2: The Role of News Trust in the Relationship between News Literacy and Fact-Checking Intent

Studies that previously examined media trust and information seeking in crises [95] found that users were more engaged in assessing the credibility of the online information they consumed. When users exist in fear-inducing environments, like the COVID-19 pandemic, they may indulge in more information seeking related to health, which translates into heightened trust in media. Additionally, the level of interaction between the person seeking information and the information-providing medium (such as the media) will probably affect how that person perceives the merits (such as credibility) of the medium, which could affect their beliefs about health risks [95]. However, these suggestions are significant for users who employ heuristic information processing for simple decision making with less cognitive effort. This indicates differences in understanding media messages and can lead to the misjudgment of information, which is why they suggest literacy as the solution for not recognizing misinformation [96].

More social media users were found to spread misinformation unwittingly on social media than those who shared it knowingly [54]. This may indicate that people who are more news-literate are less likely to spread misinformation knowingly on social media. One of the reasons may be that news literacy may cause a higher level of cautiousness towards news on social media [54,97,98]. This may further indicate that cautiousness results in some level of critical thinking and skepticism, which play a role in the intention to accept, reject, or share misinformation [58,99]. Supporting this thought, recent research suggests that trust may be necessary to a certain degree, as a “critical assessment of media practice facilitates resilience to misinformation” [98] (p. 37). If we understand critical thinking as the attitude toward the content [66], i.e., news trust, examining its role, with news literacy on one side and fact-checking intent on the other side, is meaningful.

## 3. Methodology

### 3.1. Study Design and Procedure

In this study, we employed a survey as a research method. An online self-administered questionnaire was developed to collect the data. Prior to the study’s implementation, the Institutional Review Board (IRB) of the Faculty of Arts at the University of Maribor, Slovenia approved the study. We also respected the ethical guidelines of the Declaration of Helsinki [100] and the Association of Internet Researchers [101].

**Figure 1:**
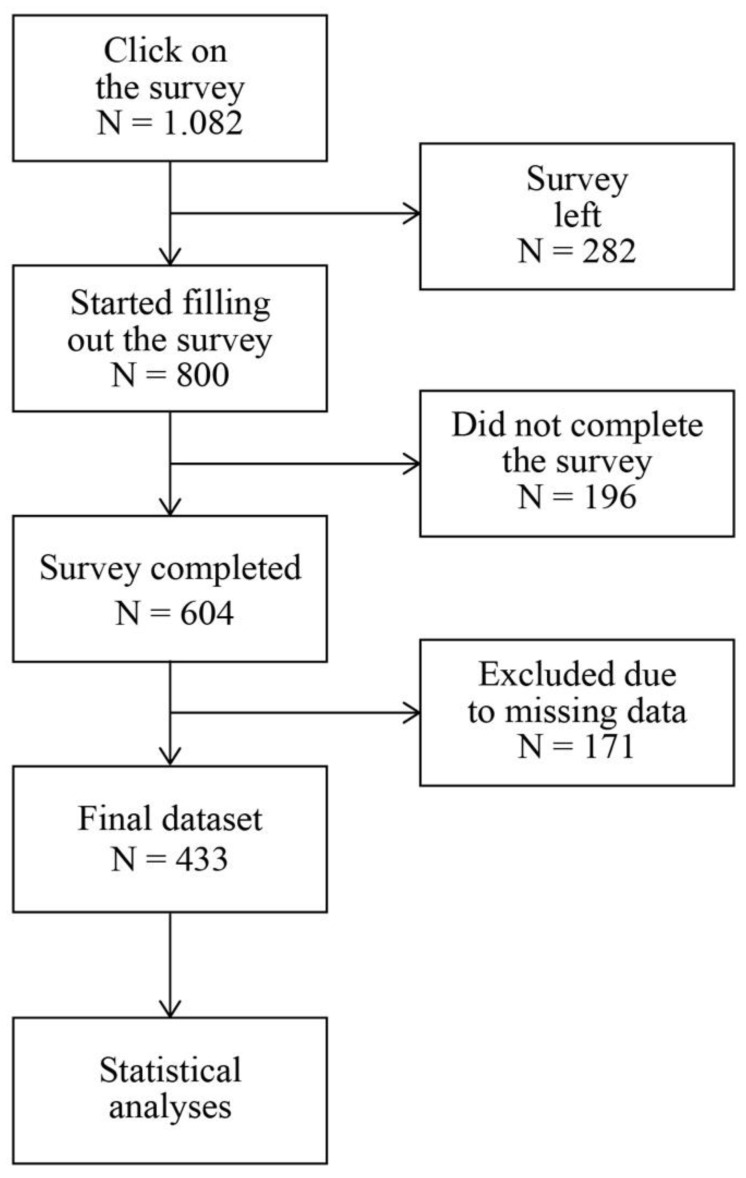
Flow chart of survey process.

The data were collected during the second wave of the COVID-19 pandemic in Slovenia. Namely, we started collecting data on 4 January 2021 and finished on 28 February 2021. To reach potential participants, we created a new Facebook profile for the purpose of gathering data. We equipped the profile with detailed information on the purpose of the study. Afterwards, we published an invitation to take part in the study on the authors’ profile on Facebook and in open and closed social media groups where the target population gathered. The focus was on groups dedicated to health communication, COVID-19, and leisure activities and groups dedicated to local communities across the country. We searched for groups using the Facebook search engine, and we also followed the algorithms’ recommendations for related Facebook groups. Figure 1 shows a flow chart of the survey process.

### 3.2. Participants

The participants were 433 adult social media users, where the majority (62.4%) were female (see Table 1). All of them were Facebook users [47], which led to us examining relevant social media platforms, as Facebook identified around 90 million pieces of content that circulated false information about COVID-19 in only two months in 2020 [102]. The participants were, on average, 33.1 years old (SD = 11.92), while participants in the 18–34-year age group prevailed (59.8%). While the age structure of Facebook users in the population can vary depending on the region and the available data are scarce, in our study, it was close to the age structure of Facebook users globally. In 2023, Facebook’s largest audience was those aged 18–34 years, accounting for 51.4% of global users [103]. Regarding education, most participants in our study had tertiary education (60%), indicating they had a Bachelor’s, Master’s, or PhD degree. Those with secondary education followed (27.3%), indicating general or vocational technical and secondary professional or technical education.

### 3.3. Measuring Instrument

Besides measuring demographic characteristics (age, education, use of social media, and COVID-19 self-experience), we measured three main concepts: news literacy, news trust, and fact-checking intent. Each was assessed using questions with response categories that had a 5-point Likert scale, ranging from 1 = “strongly disagree” to 5 = “strongly agree”.

News literacy was measured using three subdimensions: (1) authors and audiences (NLauthor), (2) messages and meanings (NLmessage), and (3) representation and reality (NLrepresent) [104]. We used 13 items, including “Creators of COVID-19 news in social media more likely chose to publish stories that could be equipped with good photos and/or videos.” News trust was measured using three subdimensions: (1) selectivity of facts (NTselect), (2) accuracy of depictions (NTaccuracy), and (3) source assessment (NTassess) [89]. Twelve items were provided, including “The information in news about COVID-19 on social media is true.” Fact-checking intent was measured using three subdimensions: (1) seeking sources (FCIseeksource), (2) seeking others’ opinions (FCIseekopinion), and (3) detecting misleading information with online tools for fact-checking (FCIdetect) [81,82,83]. We used 12 items, including “The next time I engage with news related to COVID-19, I plan to use web sites specialized in detecting incorrect information.”

The measuring instrument proposed in the current study relied on existing research [81,82,83,89,104], where a comprehensive construction procedure was followed. The measuring instrument’s content was developed according to the concepts playing a role in the conceptual model, considering the findings of the literature review and the research gap recognized in the meantime. The measures that were recognized to be helpful for measuring the concepts in this study were adapted to the context of the current research. To ensure the face validity of the measuring instrument, the initial item pool was reviewed by an expert panel consisting of five expert researchers in the field of Media Communication. The item pool was refined, and redundant, ambiguous, or irrelevant items were eliminated. Afterward, a pilot test with a sample of five participants was conducted, and the items were revised based on their feedback. Then, a pretest with a panel of five expert researchers in the field of Media Communication was conducted once again. The final measuring instrument was developed based on the feedback.

### 3.4. Data Reliability and Validity Analysis

The data analysis started with data screening, followed by a confirmatory factor analysis. The analysis confirmed the three-dimensional structure of the constructs news literacy and fact-checking intent, while news trust turned out to be a two-dimensional instead of three-dimensional construct, consisting of the dimensions NTaccuracy and NTselect (see Table 2). Due to convergent validity issues, we dropped the items with factor loadings lower than 0.5 [105]: four items in the variable ‘NLmessage’ as well as one item in the variables ‘NLauthor’, ‘NLrepresent’, and ‘NTaccuracy’. An additional analysis demonstrated that the extracted factors’ internal consistency was sufficient. The Cronbach’s alpha coefficients surpassed the minimum acceptable alpha of 0.65 [106] (see Table 2). Accordingly, the model fit was achieved (see Table 3). The only marginal value was the GFI value, which could still be deemed acceptable.

Moreover, we inspected the validity and reliability of the model using the Composite Reliability (CR), the Average Variance Extracted (AVE), and the factor correlation matrix (see Table 4). The results were as follows: NLrepresent (CR = 0.718, AVE = 0.575), NTselect (CR = 0.863, AVE = 0.621), NTaccuracy (CR = 0.888, AVE = 0.531), FCIdetect (CR = 0.962, AVE = 8.893), FCIseeksource (CR = 0.929, AVE = 0.814), FCIseekopinion (CR = 0.954, AVE = 0.874), NLmessage (CR = 0.704, AVE = 0.543), and NLauthor (CR = 0.765, AVE = 0.522). The model had no reliability concerns, as the CR values exceeded the minimum recommended value of 0.7 [107]. Likewise, we found no convergent validity issues, as the AVE values exceeded the minimum recommended value of 0.5 [107]. In terms of discriminant validity, there were also no concerns [105,107]. Finally, using Harman’s single-factor test, we examined a typical approach bias. The results revealed no concerns since a single factor did not account for the majority of the variance in the model [108].

### 3.5. Structural Equation Modeling

IBM SPSS Statistics 27.0 and AMOS 27.0 were used for the structural equation modeling (SEM) process. We inspected the linear correlation between the items inside each construct before running the final structural equation model, and no issues were found.

A test of the model fit followed. All the values matched the recommended values (see Table 3 column “Recommended value”). The only marginal value was the GFI value, which indicated that the overall model fit was acceptable (X2 = 694.707, DF = 312, Cmin/df = 2.227, RMSEA = 0.053, GFI = 0.891, NFI = 0.914, CFI = 0.951). Finally, we tested the validity and reliability of the model again, and the values for AVE and CR were generally acceptable. There were some minor discrepancies for the constructs ‘NLrepresent’ (CR = 0.595, AVE = 0.329), ‘NLmessage’ (CR = 0.534, AVE = 0.287), and ‘NLauthor’ (CR = 0.581, AVE = 0.428).

## 4. Results

**Figure 2:**
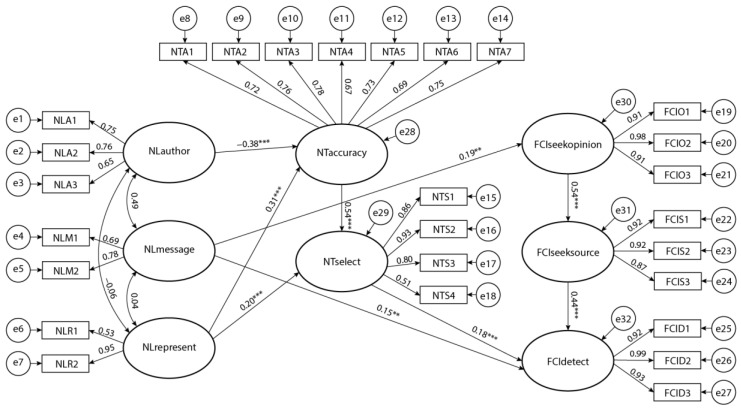
The model resulting from the path analysis (significance levels: ** *p* < 0.01; *** *p* < 0.001).

Figure 2 shows the results, where the links between variables are accompanied by path coefficients that are marked when statistically significant. Twenty-five percent of the variability in the final dependent variable ‘FCIdetect’ was explained by the latent variables.

The model suggested a negative moderate statistically significant effect of ‘NL author’ on ‘NTaccuracy’. It indicated that the higher the degree of news literacy in terms of awareness that the authors of social media news may influence the content, the lower the degree of news trust in terms of the belief that COVID-19 news on social media accurately depicts reality and what is actually happening. The latter further moderately affected the variable ‘NTselect’. This indicated that the more users believed that reality was presented accurately in the news, the more they believed that social media news included all the essential information, facts, and points of view about COVID-19. Moreover, we found that there was a moderate statistically significant effect of ‘NTselect’ on ‘FCIdetect’. This indicated that the more users believed that all essential information was included in the news, the higher the intention to use online tools for fact-checking. Intriguingly, we found a weak positive statistically significant effect of ‘NLmessage’ on ‘FCIseekopinion’ and ‘FCIdetect’. This indicated that the more users were aware that the news creators used various techniques to depict COVID-19, the more they intended to check other users’ opinions about these news creators and the more they intended to use online tools to fact-check information. Likewise, we found a moderate positive statistically significant effect between the variables ’FCIseekopinion’ and ‘FCIseeksource’ as well as between ‘FCIseeksource’ and ‘FCIdetect’. These results indicated that the more users intended to check other users’ opinions about news creators, the more they intended to seek other sources of information and, finally, use online tools for fact-checking. Finally, we found weak effects of ‘NLrepresent’ on ‘NTaccuracy’ and ‘NTselect’. This indicated that the more users were aware of news literacy in terms of the awareness that messages represented the reality of COVID-19, the more they trusted the accurate presentation of COVID-19 and the selective inclusion of essential information in the news.

## 5. Discussion

The aim of the present study was to examine how fact-checking intent regarding news about COVID-19 posted by users, rather than news organizations, on social media was explained by news literacy and news trust. Firstly, the results showed that the lower the awareness that the post including news depended mainly on the news creator’s selectivity of the topic, the higher the belief that the COVID-19 situation was presented accurately in the post. These findings support the existing research on social media interactions, which may increase the misperception of news when engaging on these platforms, which serve as types of risk-attenuation stations [109]. Additionally, we found that when users believed that all essential information, facts, and viewpoints about COVID-19 were presented, they had greater trust that the news delivered an accurate picture. As a result, surprisingly, they intended to use online fact-checking tools to a greater extent to verify the information in the posts on COVID-19.

Prior research [63,110] suggested that even those with a high level of news literacy may not apply their knowledge to differentiate between low- and high-quality information. Likewise, our findings indicate that even when social media users do not question themselves much about news creators, they still develop news trust but, nevertheless, check the information they recruit. This is in line with prior research [64], which revealed that news trust grew significantly during the recent international health crisis of COVID-19. This could have been due to users’ perceptions of other users’ understanding of online media and information dissemination, resulting in striving to stop others from spreading false information through their network because they had a high moral sense for the well-being of society at large [20]. Accordingly, users used social media as a source of information about COVID-19 and engaged with the news. When they felt they were being informed, they still sought more information and checked the accuracy of this news. This could complement a finding in the prior research [97], where users with a higher level of news literacy demonstrated cautiousness towards the news on social media.

On the contrary, our findings indicate that caution towards information on social media does not necessarily depend on a particular level of news literacy. It is plausible that some other factors exist, such as the perception of the risk [109] of acquiring COVID-19. Namely, another previous study [111] substantiated the positive relationship between news exposure and risk perception. Thus, more research is needed to explore these social media users’ habits fully and perhaps introduce various media types that users employ to get informed about health-risk topics.

A similar explanation may apply to our second finding. The results revealed that the more users were aware that news might affect their opinion about COVID-19, the more they believed that the news accurately portrayed the actual situation related to COVID-19 and that all essential information, facts, and points of view related to COVID-19 were included. As a result, they intended to use online fact-checking tools to check the information they encountered about COVID-19. This finding suggests that a higher level of news literacy was associated with a higher level of news trust [50] and, finally, with a higher intention to use fact-checking tools [56,57]. It is plausible that users are in a social media bubble and are, thus, exposed to news selectively from sources in their social network [112], especially politics-infused ones [94]. This may have resulted in their news trust, but, concurrently, a particular level of cautiousness was still evident [97]. As this study dealt with news published on social media not by news organizations but rather by other users, our findings suggest that users trust information about COVID-19 on social media but are still ready to hear a different opinion when it exists. An additional study would be needed to clarify who users retrieve information about COVID-19 from on social media. This would allow the differentiation of the effects of various sources, i.e., government, non-governmental, and naïve users.

Thirdly, similar to existing research about user fact-checking behavior [43,52], we found that the more users were aware that the news creators used various techniques to depict COVID-19, the more they intended to check other users’ opinions [54] of these news creators and use online tools for fact-checking. As most of our respondents were relatively young (aged up to 34 years) and probably used social media actively, it is plausible that they were skilled Information and Communication Technology (ICT) users, understanding how news for social media is produced and disseminated [43]. This finding is congruent with previous studies, where it was found that high-news-literacy students were shown to be more inclined to seek out news for socializing than their low-news-literacy counterparts [113].

The convenience sample is the primary drawback of the current study. The data collection was limited, as advertising policies did not allow the survey about COVID-19 to be publicized [114,115]. However, we were interested primarily in social media users rather than the general population in Slovenia. Our case calls for further actions and an update of social media policies when collecting data on sensitive topics for scientific purposes. Another limitation of our study is derived from the demographic characteristics of the sample, which was biased by not representing the population statistically, as well as the gender imbalance in favor of women.

## 6. Conclusions

The present study highlights news literacy, trust, and verifying the news by answering how users perform fact-checking in a misinformation-prone social media environment during a health crisis. Misinformation during COVID-19 increased the need for information verification awareness for content providers and audiences [116]. Fact-checking and verification services for online information can, thus, be beneficial during a health crisis and when fighting online misinformation, for instance, in a democracy [117]. The main contribution of the present study is, therefore, findings with potentially broader use, as we concluded that news literacy affects news trust, and both finally explain fact-checking intent, offering an attempt at a holistic assessment of the value of news literacy in combating misinformation, as called for in previous research [4,40,44]. Namely, our study stresses that users’ awareness of the quality of both the author and the message is essential for users to develop trust in news content, check information, and retrieve accurate information supported by previous findings [55,56,57,58].

Focusing on the audience group, our findings, firstly, revealed that those who demonstrated higher levels of news literacy at the level of the content (how media representations influence perceptions of reality) also developed higher levels of news trust. Consequently, they intended to check or verify the information they encountered to a greater extent by using online tools for fact-checking. Secondly, those less aware of news creators and their influence also, intriguingly, developed higher levels of news trust but, finally, still, to a greater extent, intended to use online tools for fact-checking. The employment of these tools was also found to be dependent on news literacy at the level of the content. Thirdly, those who demonstrated higher levels of news literacy at the level of the content (how meanings in messages are conveyed and how messages can influence the user) demonstrated higher intention to use online fact-checking tools and seek other users’ opinions to verify information. Therefore, being news-literate at the level of the content leads to the multidimensional checking/verifying of information. On the contrary, being news-literate at the level of the authors leads to a more narrowly focused checking/confirming of information on social media.

Our study has theoretical as well as practical implications. The findings on media literacy and news trust can be a foundation for researchers who focus their studies on examining misinformation on social media and even those who create courses, guidelines, and news-literacy-based educational frameworks for users since the effectiveness of educational efforts was under question [83]. Secondly, the present study has provided a research framework regarding users’ characteristics, attitudes, and intentions towards actions on social media [54]. In practice, our findings about how social media users perceived news about COVID-19 may serve professional communicators in health crises or other similar crises. They may allow them to develop a more efficient communication strategy for social media users.

Our study does not come without opportunities for improvement. Future studies may include more variables within the concepts of news literacy, news trust, or verification actions relevant to various situations. Another opportunity is to spread the research across other social media platforms, as was the case in previous studies [2,6,54], and the range of crisis topics is up to researchers themselves. It would also be intriguing to develop a model where researchers would consider multiple types of entities who share COVID-19 news on social media, i.e., government sources, non-governmental sources, naïve users, etc., and compare the findings, confronting their news literacy level, news trust, and willingness to take action through fact-checking intent or even other similar media verification and source-related activities.

## Figures and Tables

**Table 1:** Baseline characteristics of study participants (*n* = 433).

Characteristic	Number (*n*)	Percentage (%)
Gender		
Female	270	62.4
Male	163	37.6
Age		
18–24 years	147	33.9
25–34 years	112	25.9
35–44 years	84	19.4
45–54 years	62	14.3
55–64 years	28	6.5
Education		
Primary (basic school)	5	1.2
Secondary (general or vocational technical and secondary professional or technical education)	168	38.8
Tertiary (Bachelor’s, Master’s, or PhD degree)	260	60

**Table 2:** Factor loadings and Cronbach’s alpha coefficients of constructs.

Construct	Abbreviation of Variable	Cronbach’s Alpha Coefficient	Item	Factor Loading
News literacy in authors and audiences	NLauthor	0.756	NLA1	0.75
			NLA2	0.77
			NLA3	0.65
News literacy in messages and meanings	NLmessage	0.702	NLM1	0.71
			NLM2	0.76
News literacy in representation and reality	NLrepresent	0.672	NLR1	0.55
			NLR2	0.92
News trust in the accuracy of depictions and source assessment	NTaccuracy	0.888	NTA1	0.72
			NTA2	0.76
			NTA3	0.78
			NTA4	0.67
			NTA5	0.73
			NTA6	0.69
			NTA7	0.75
News trust in the selectivity of facts	NTselect	0.846	NTS1	0.85
			NTS2	0.93
			NTS3	0.79
			NTS4	0.50
Fact-checking intent in seeking others’ opinions	FCIseekopinion	0.961	FCIO1	0.91
			FCIO2	0.98
			FCIO3	0.92
Fact-checking intent in seeking sources	FCIseeksource	0.928	FCIS1	0.92
			FCIS2	0.92
			FCIS3	0.87
Fact-checking intent in detecting misleading information	FCIdetect	0.952	FCID1	0.92
			FCID2	0.99
			FCID3	0.93

**Table 3:** The overall fit of the model.

Notation	Recommended Value	Calculated Value
Chi-square value (X2)		652.134
Degrees of freedom (DF)		296
Chi-square value/degrees of freedom (Cmin/df)	≤3.0	2.203
Root-mean-square error of approximation (RMSEA)	≤0.10	0.053
Goodness-of-fit index (GFI)	≥0.90	0.896
Normed fit index (NFI)	≥0.90	0.920
Comparative fit index (CFI)	≥0.90	0.954

**Table 4:** Factor correlation matrix.

Abbreviation	NLrepresent	NTselect	NTaccuracy	FCIdetect	FCIseeksource	FCIseekopinion	NLmessage	NLauthor
A	**0.758**							
B	0.379	**0.788**						
C	0.338	0.600	**0.729**					
D	0.165	0.184	0.113	**0.945**				
E	0.106	0.037	−0.036	0.448	**0.902**			
F	0.058	0.072	−0.046	0.443	0.537	**0.935**		
G	0.030	−0.010	−0.154	0.186	0.102	0.166	**0.737**	
H	−0.057	−0.248	−0.401	0.019	0.123	0.124	0.497	**0.722**

The diagonal components in bold are the square roots of AVE. A = NLrepresent, B = NTselect, C = NTaccuracy, D = FCIdetect, E = FCIseeksource, F = FCIseekopinion, G = NLmessage, H = NLauthor.

## Data Availability

The raw data supporting the conclusions of this article are freely available at https://github.com/ineskozuh/kozuh-caks-2023 (accessed on 4 August 2023).
